# Supplementary figures and images for: Targeting plasmid-encoded proteins that contain immunoglobulin-like domains to combat antimicrobial resistance
Source: eLife. 2024 Jul 24;13:RP95328. doi: 10.7554/eLife.95328 (PMC11268884; doi:10.7554/eLife.95328)

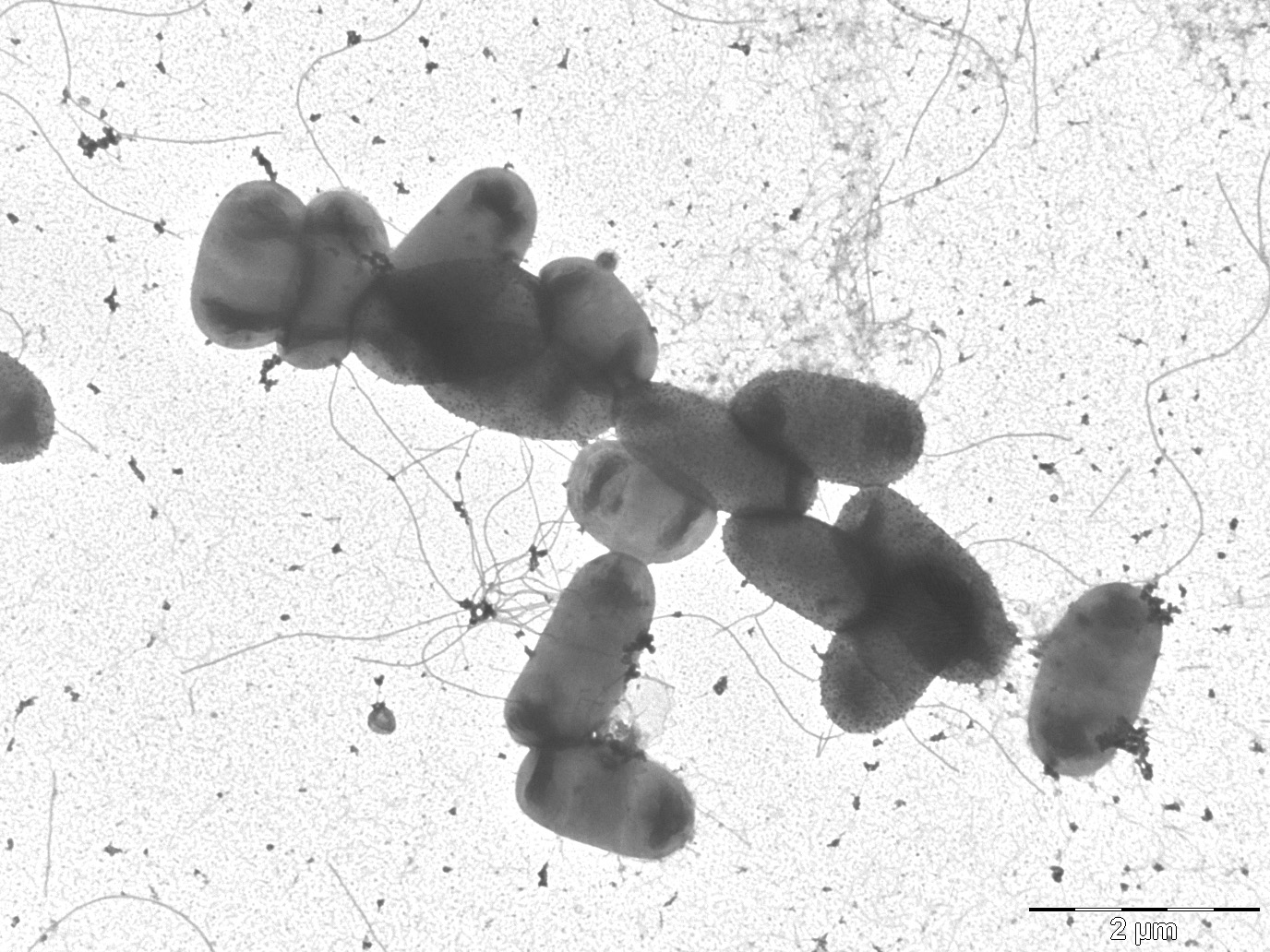

Supplement: Figure 7—source data 1. [file elife-95328-fig7-data1.zip › imagenes figura 7/SV5015-R27-dil800-9.jpg]

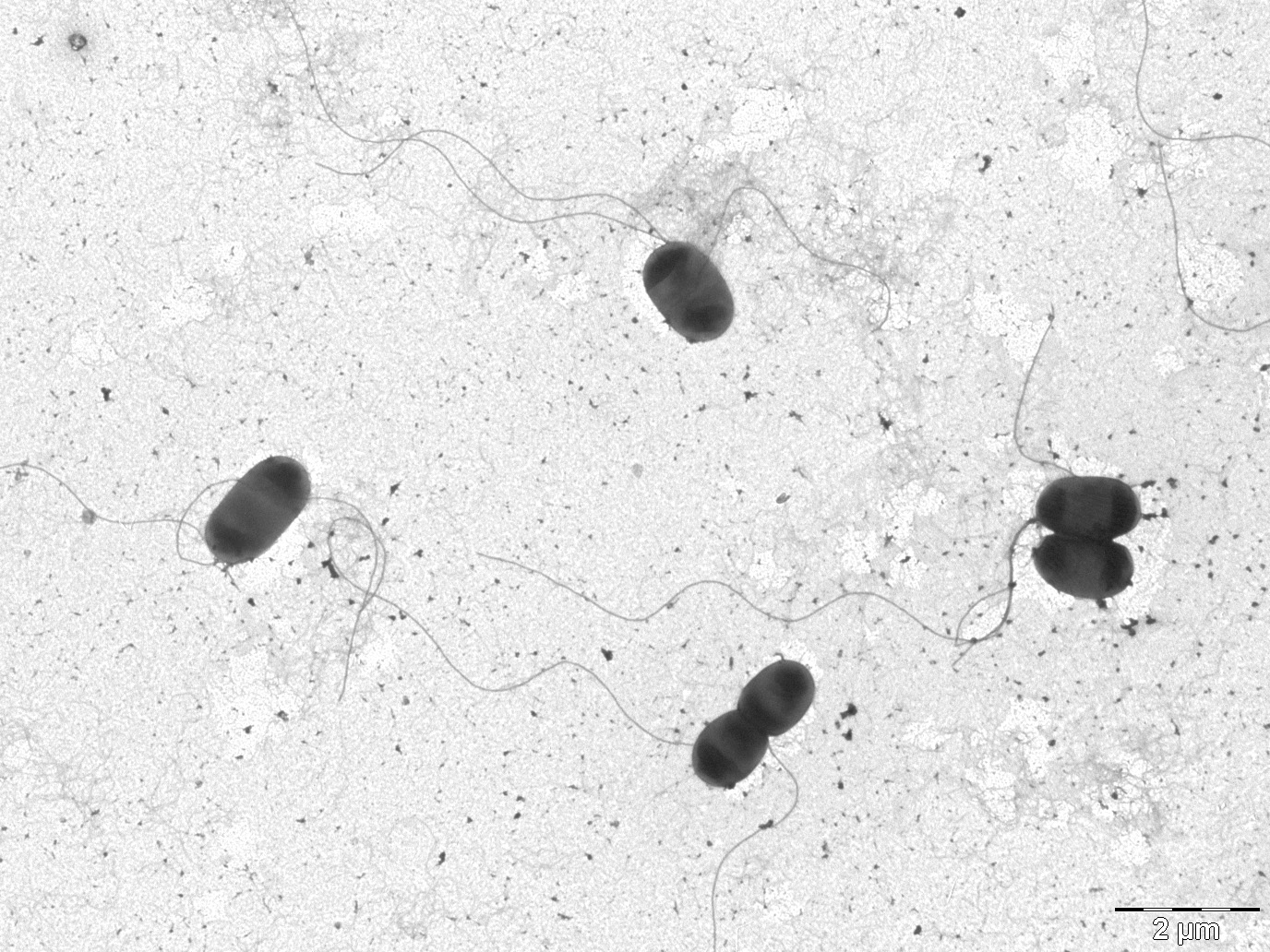

Supplement: Figure 7—source data 1. [file elife-95328-fig7-data1.zip › imagenes figura 7/SV5015-dil800-7.jpg]

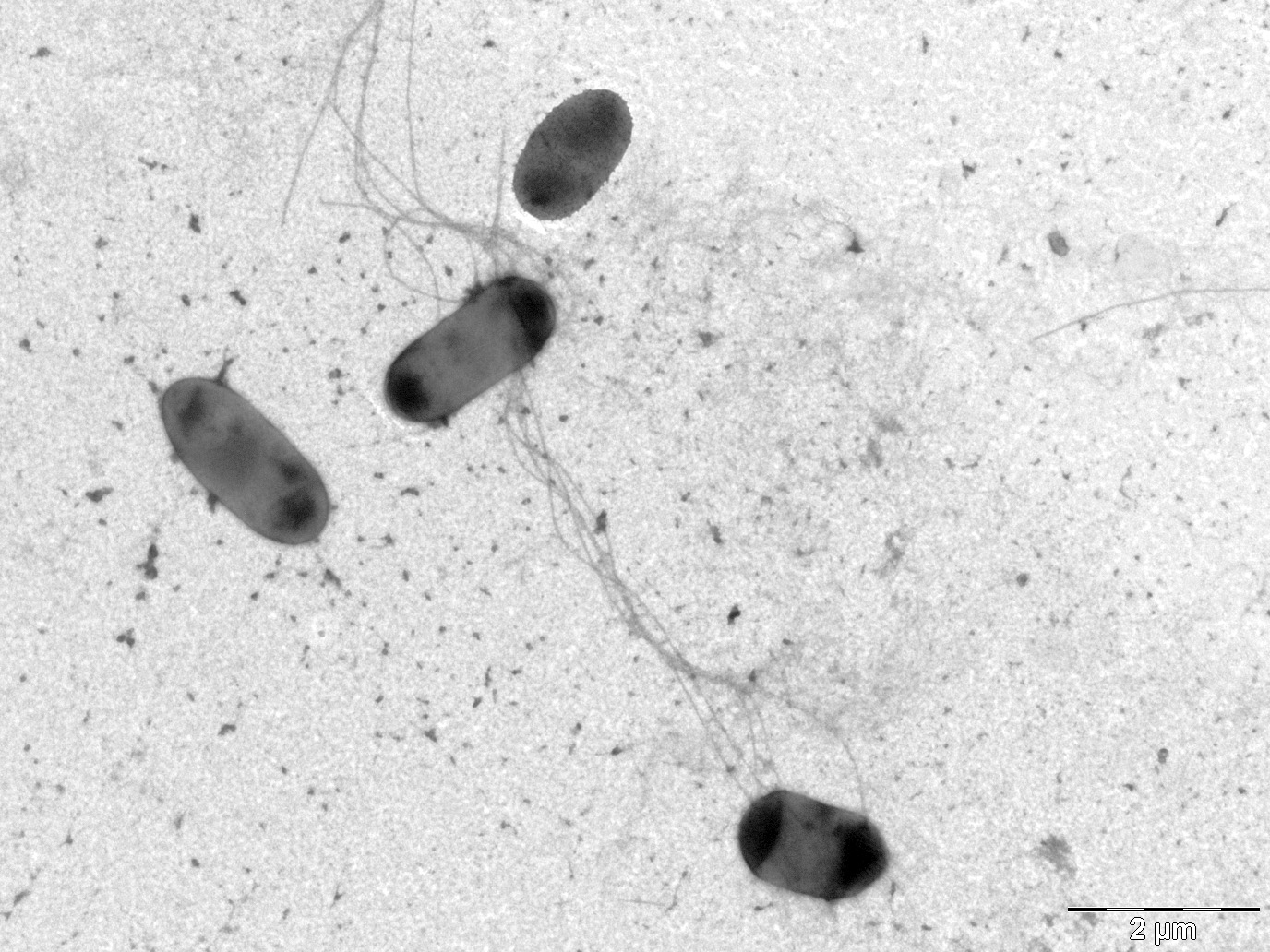

Supplement: Figure 7—source data 1. [file elife-95328-fig7-data1.zip › imagenes figura 7/SV5015-R27Mutant-dil800-1.jpg]

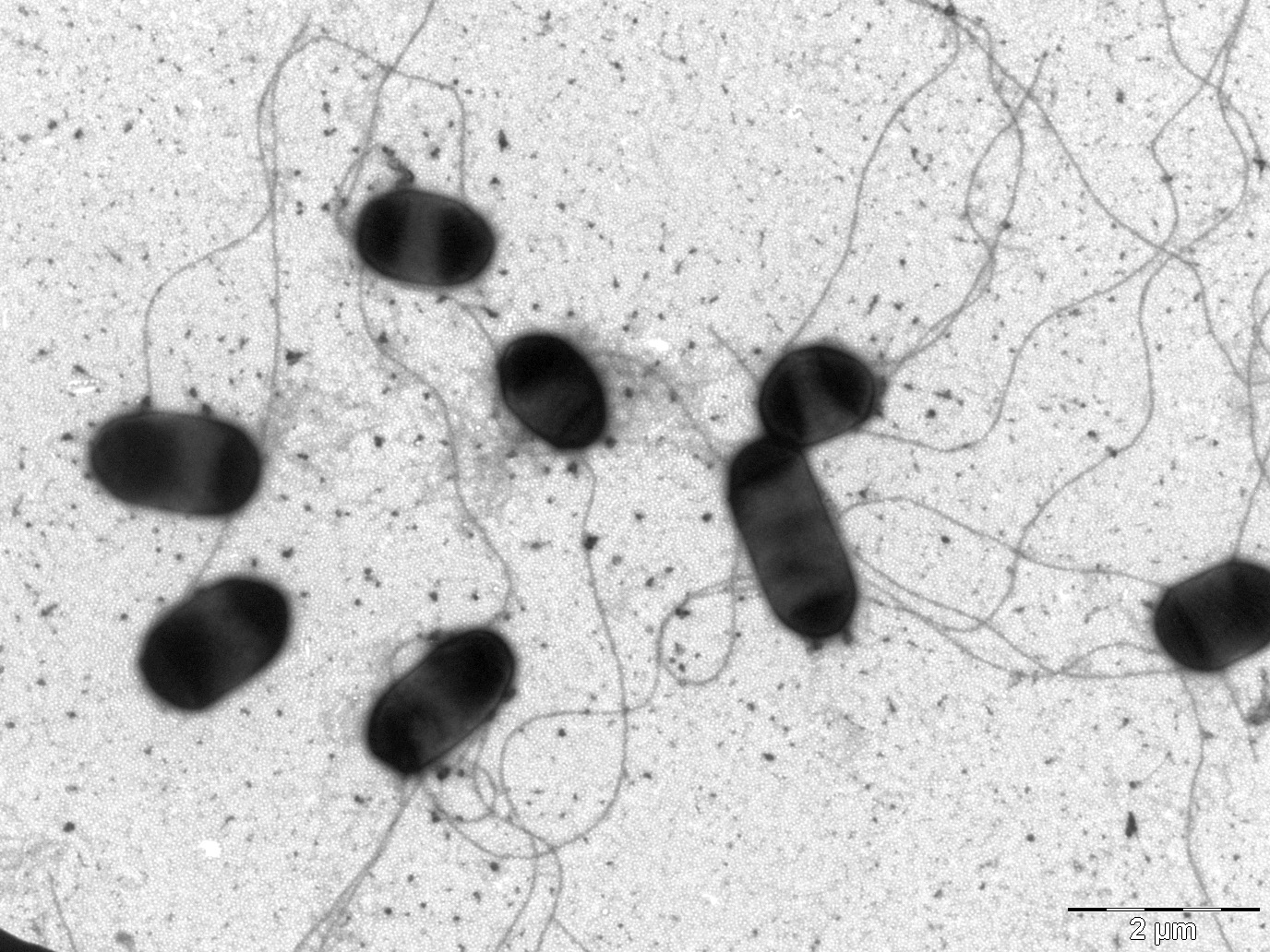

Supplement: Figure 7—source data 1. [file elife-95328-fig7-data1.zip › imagenes figura 7/SV5015-R27Mutant-dil800-3.jpg]

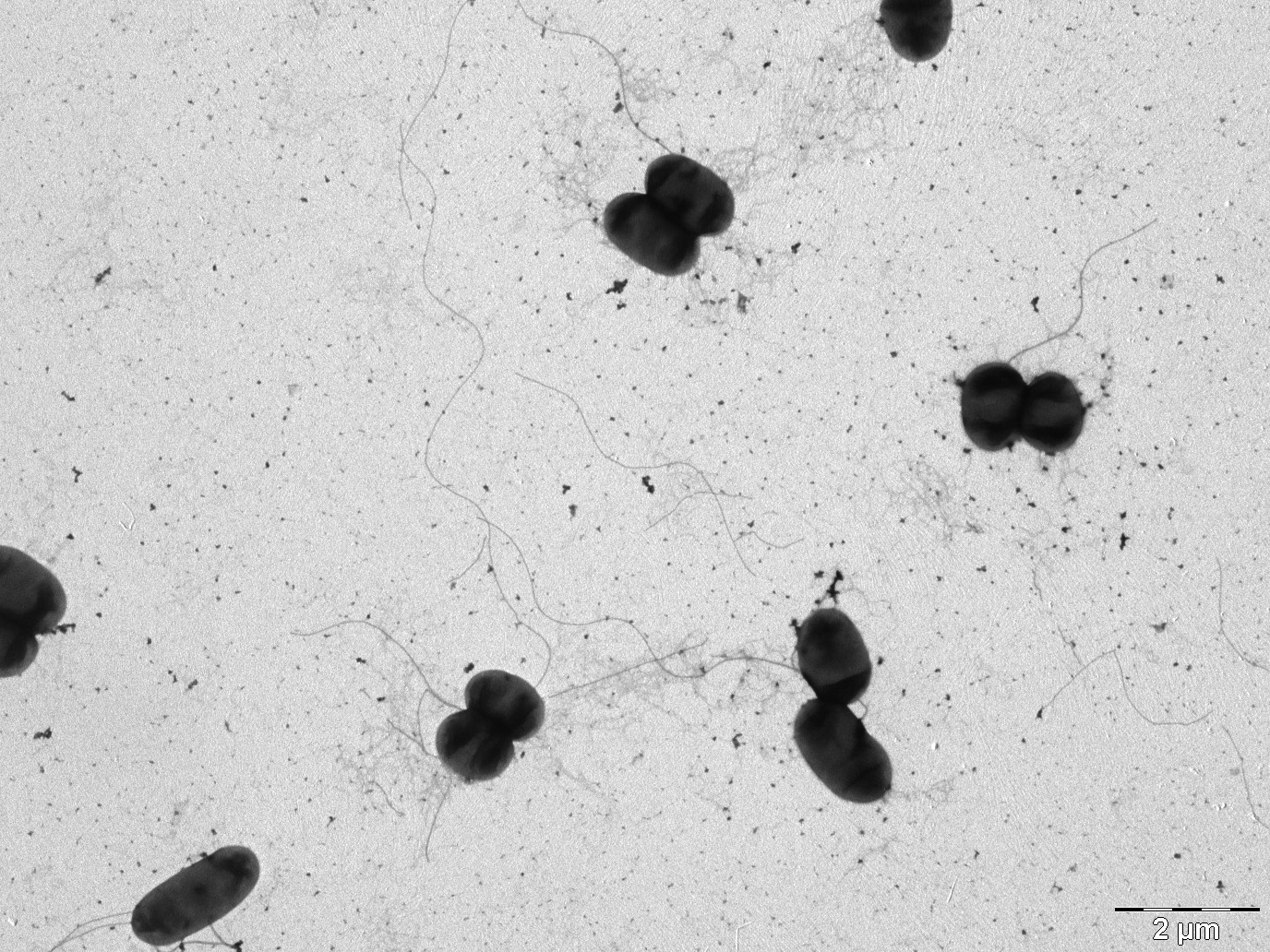

Supplement: Figure 7—source data 1. [file elife-95328-fig7-data1.zip › imagenes figura 7/SV5015-dil800-5.jpg]

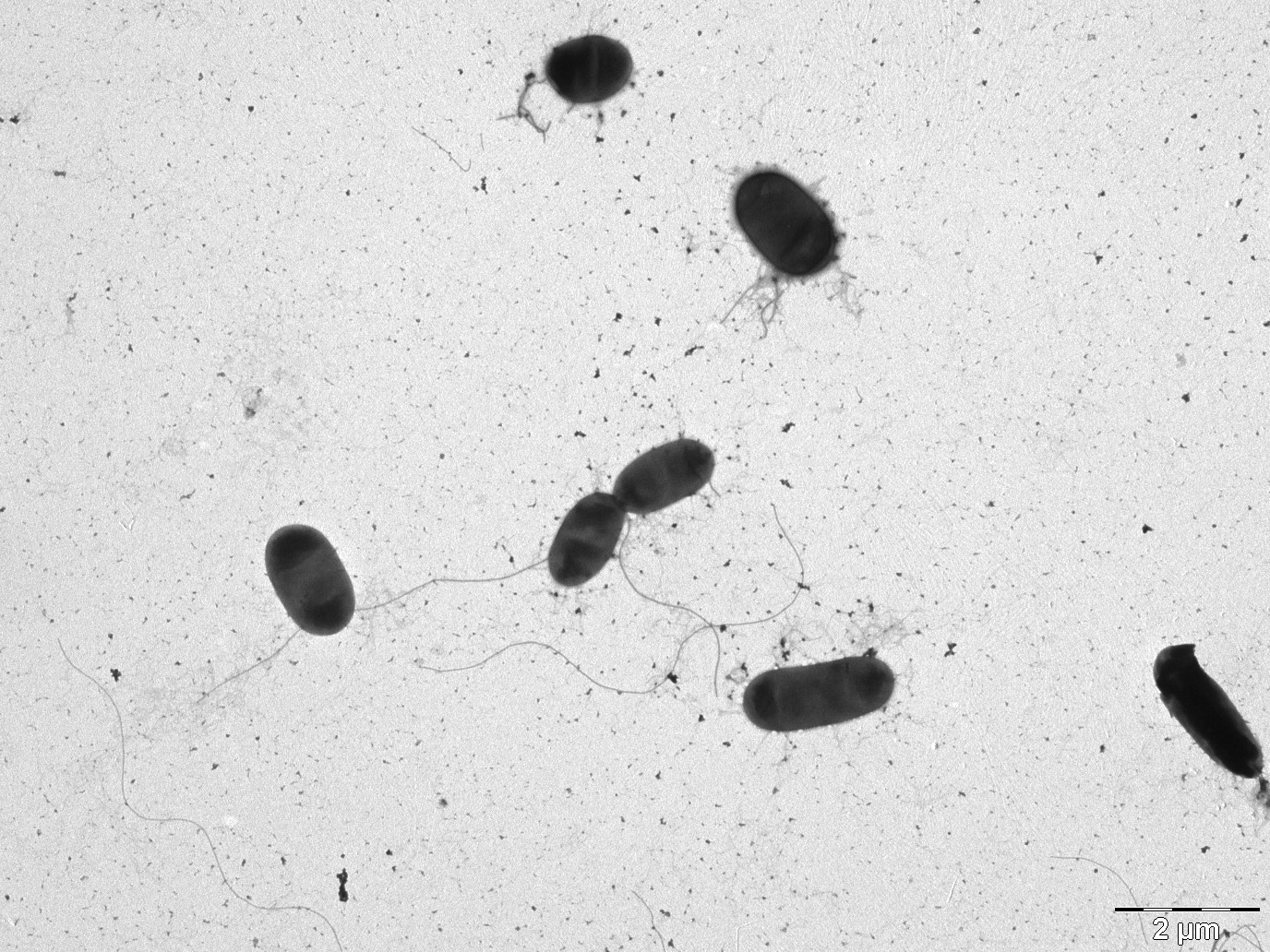

Supplement: Figure 7—source data 1. [file elife-95328-fig7-data1.zip › imagenes figura 7/SV5015-dil800-4.jpg]

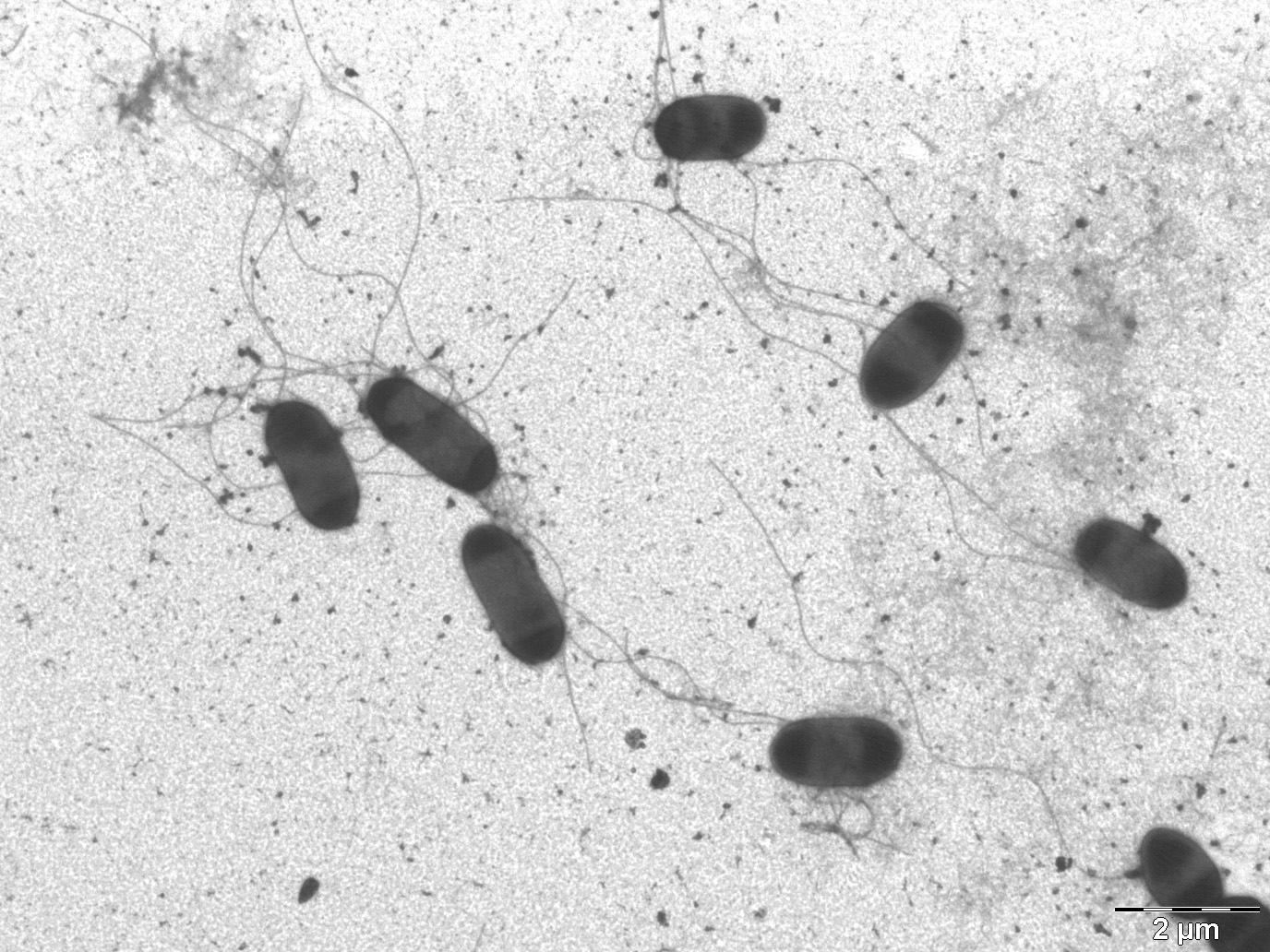

Supplement: Figure 7—source data 1. [file elife-95328-fig7-data1.zip › imagenes figura 7/SV5015-R27Mutant-dil800-2.jpg]

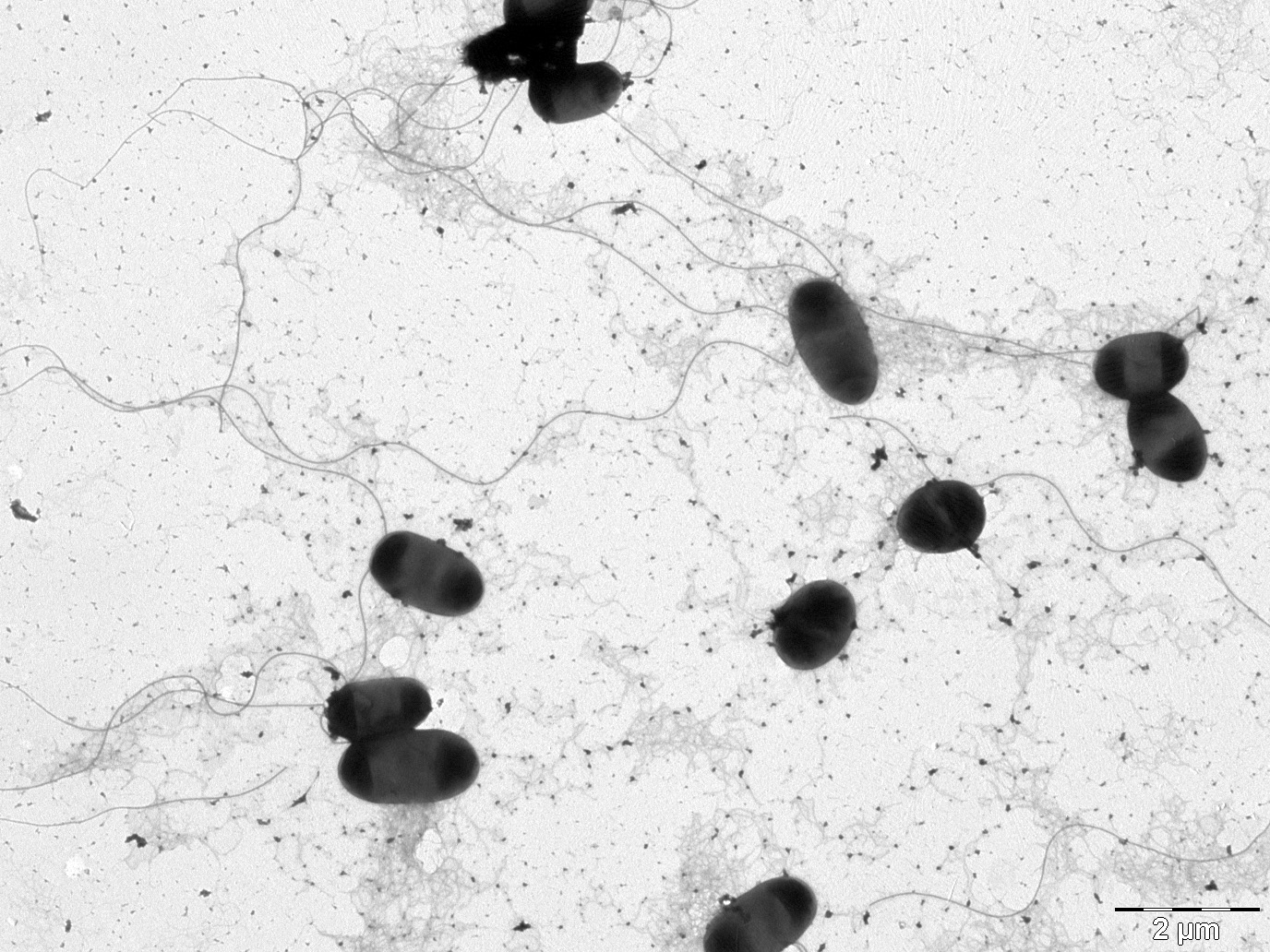

Supplement: Figure 7—source data 1. [file elife-95328-fig7-data1.zip › imagenes figura 7/SV5015-dil800-15.jpg]

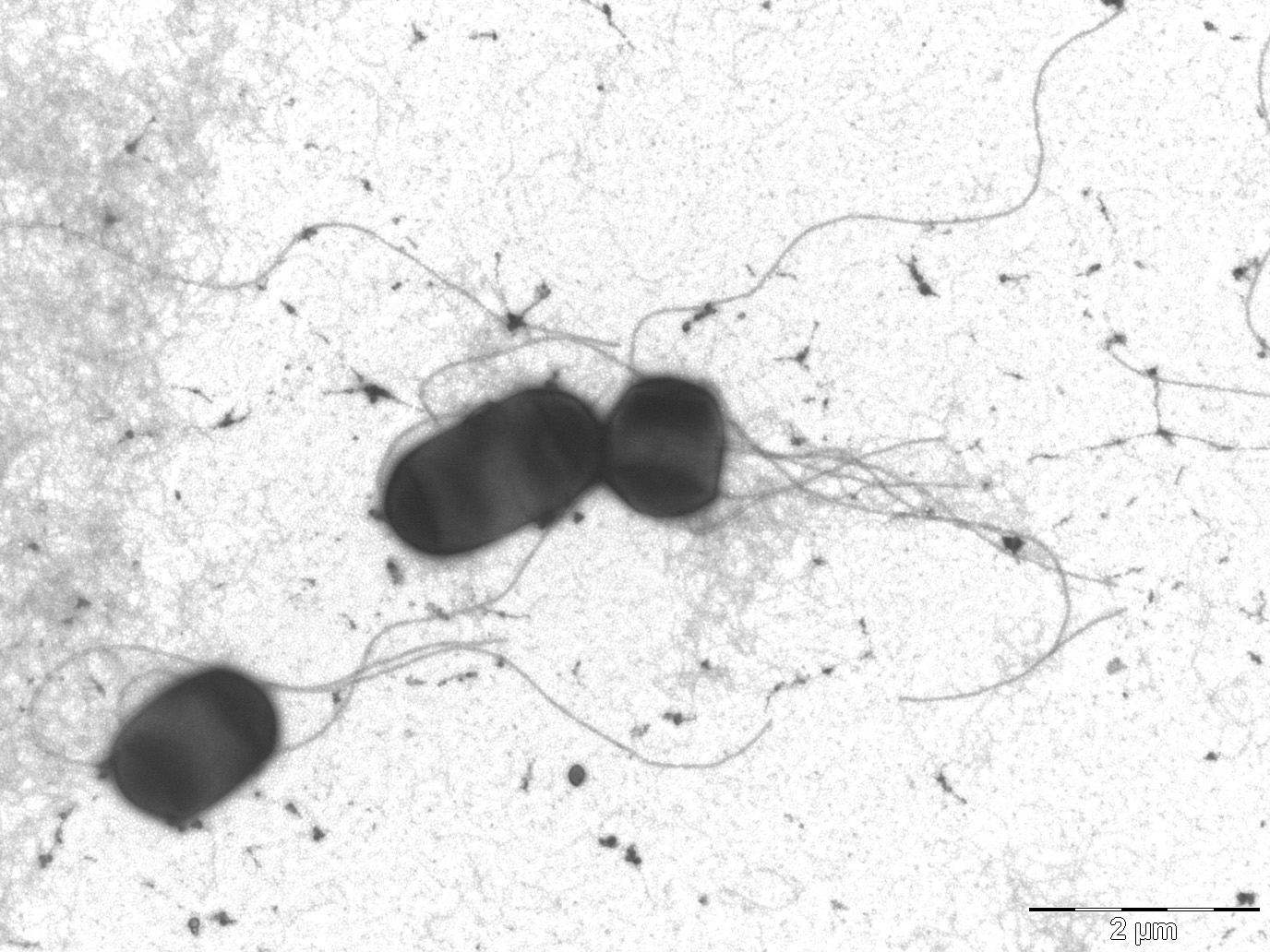

Supplement: Figure 7—source data 1. [file elife-95328-fig7-data1.zip › imagenes figura 7/SV5015-R27Mutant-dil800-6.jpg]

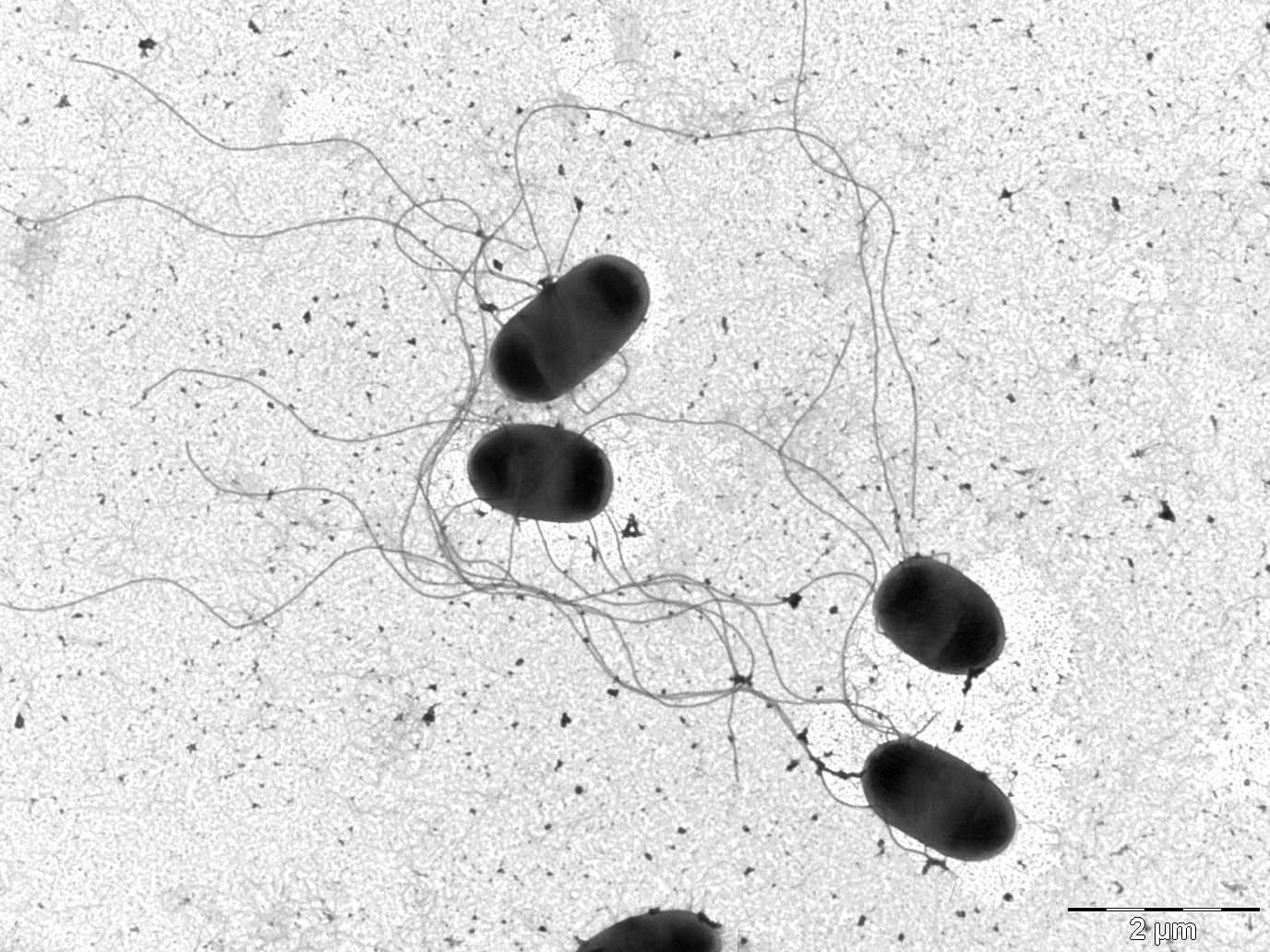

Supplement: Figure 7—source data 1. [file elife-95328-fig7-data1.zip › imagenes figura 7/SV5015-dil800-12.jpg]

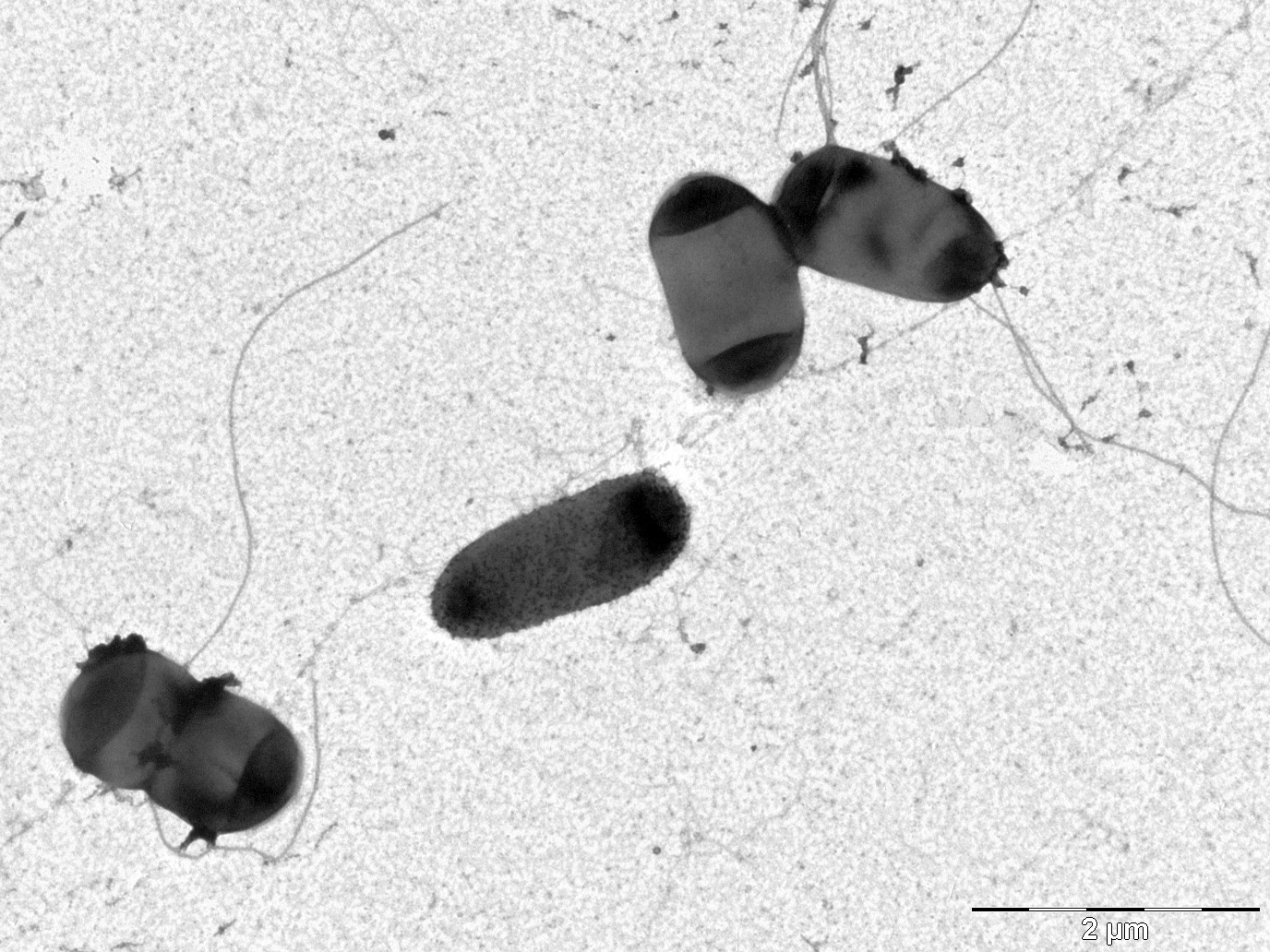

Supplement: Figure 7—source data 1. [file elife-95328-fig7-data1.zip › imagenes figura 7/SV5015-R27Mutant-dil800-5.jpg]

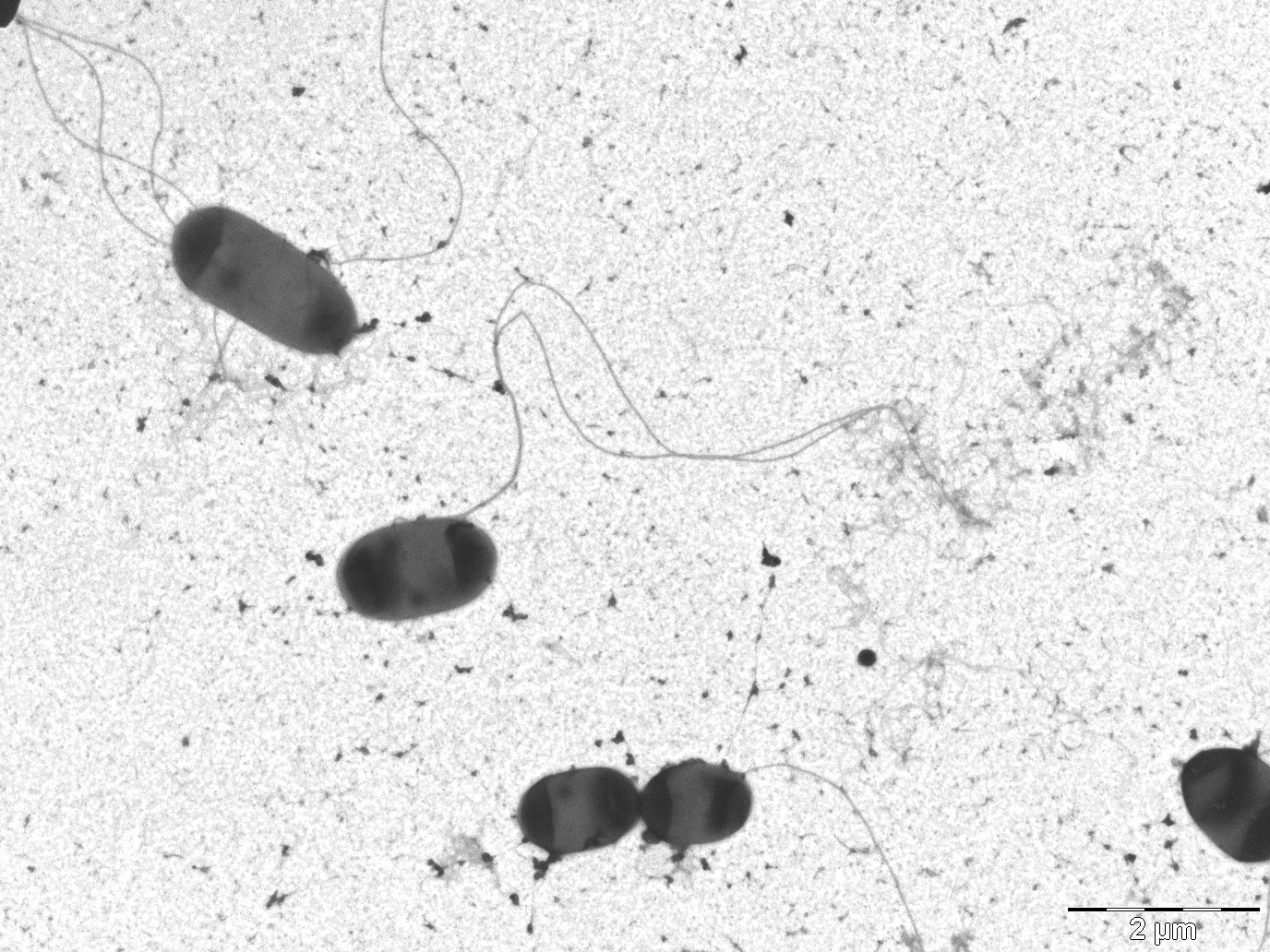

Supplement: Figure 7—source data 1. [file elife-95328-fig7-data1.zip › imagenes figura 7/SV5015-R27Mutant-dil800-4.jpg]

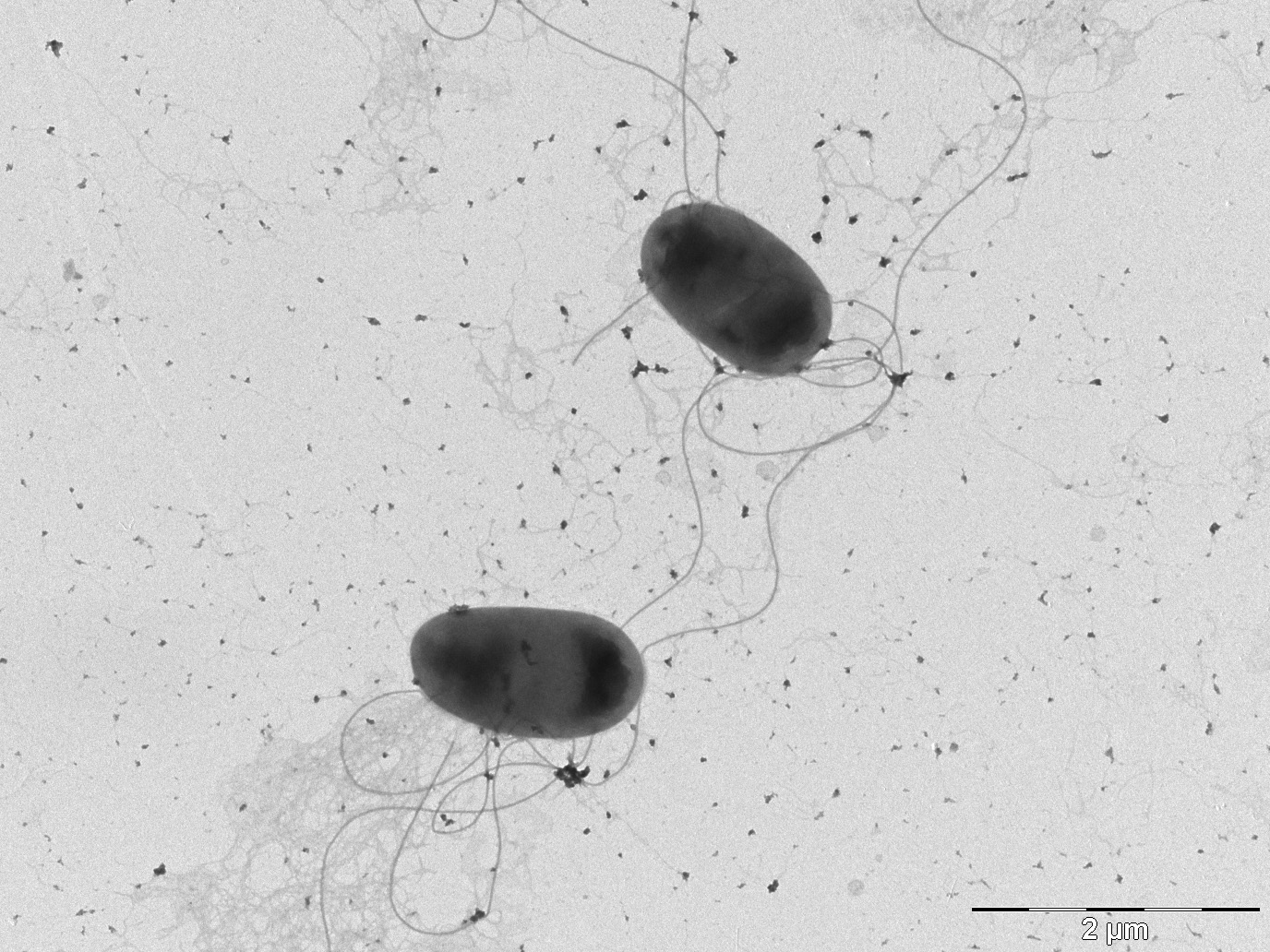

Supplement: Figure 7—source data 1. [file elife-95328-fig7-data1.zip › imagenes figura 7/SV5015-dil800-2.jpg]

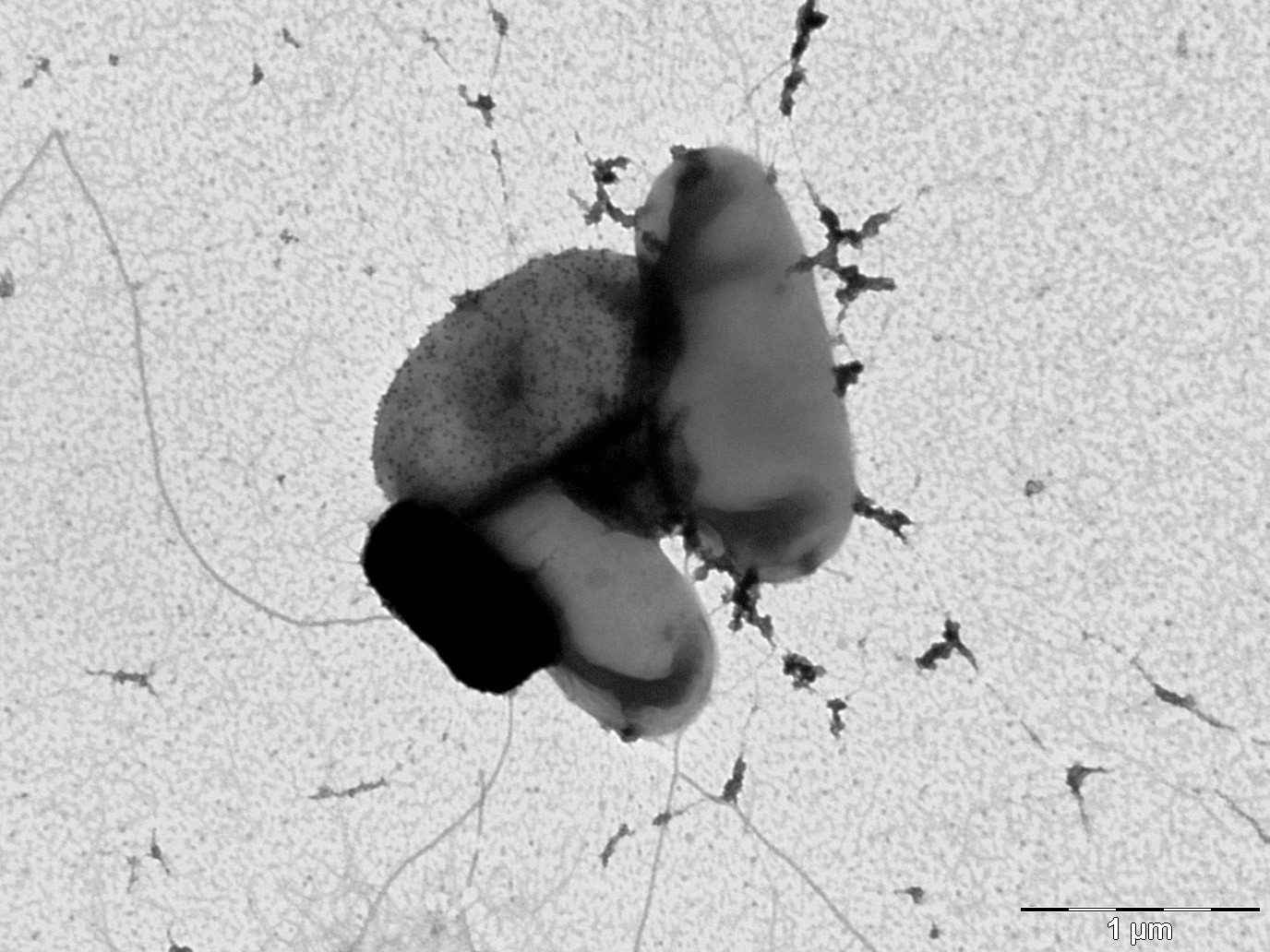

Supplement: Figure 7—source data 1. [file elife-95328-fig7-data1.zip › imagenes figura 7/SV5015-R27-dil800-4.jpg]

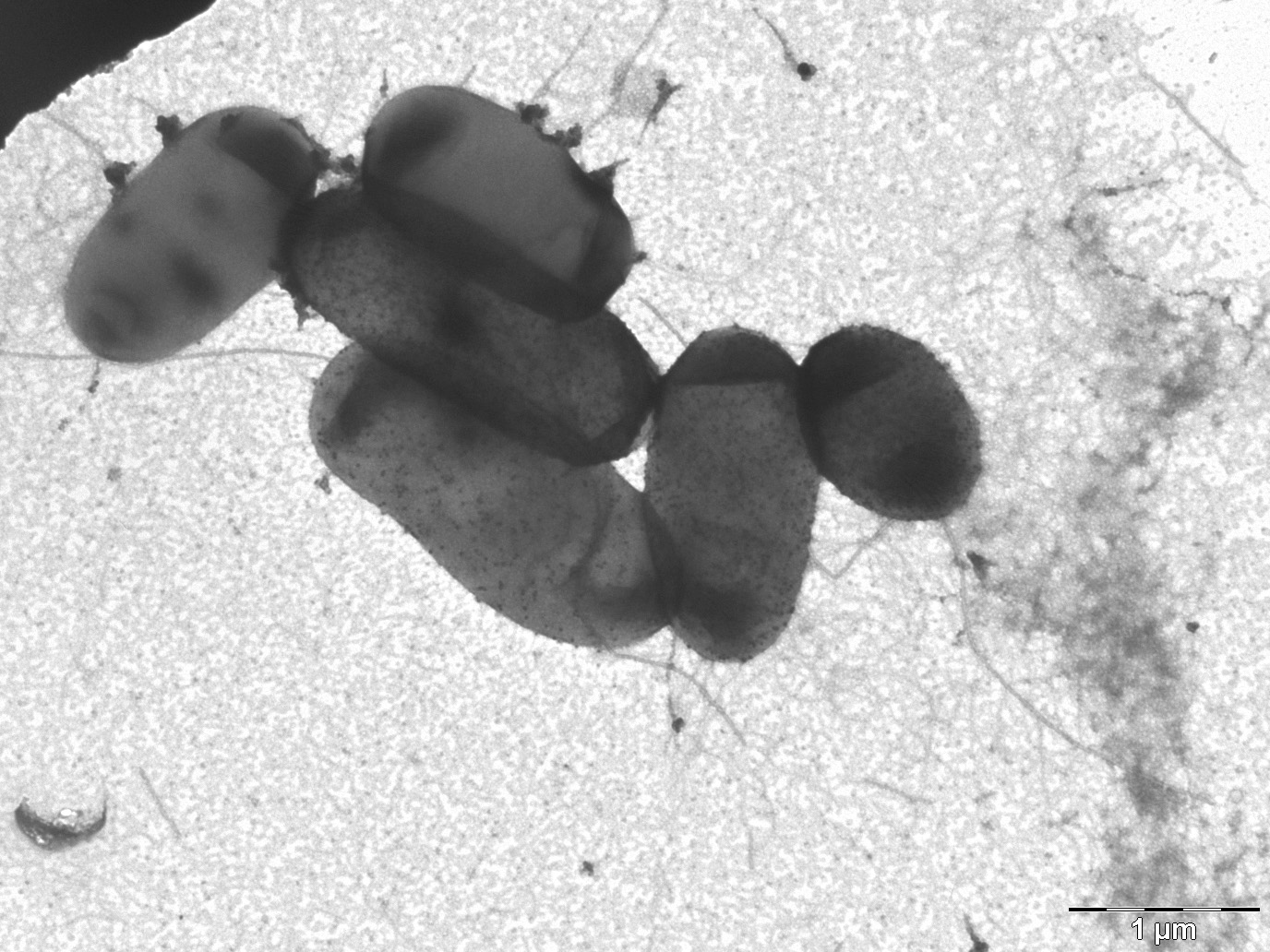

Supplement: Figure 7—source data 1. [file elife-95328-fig7-data1.zip › imagenes figura 7/SV5015-R27-dil800-5.jpg]

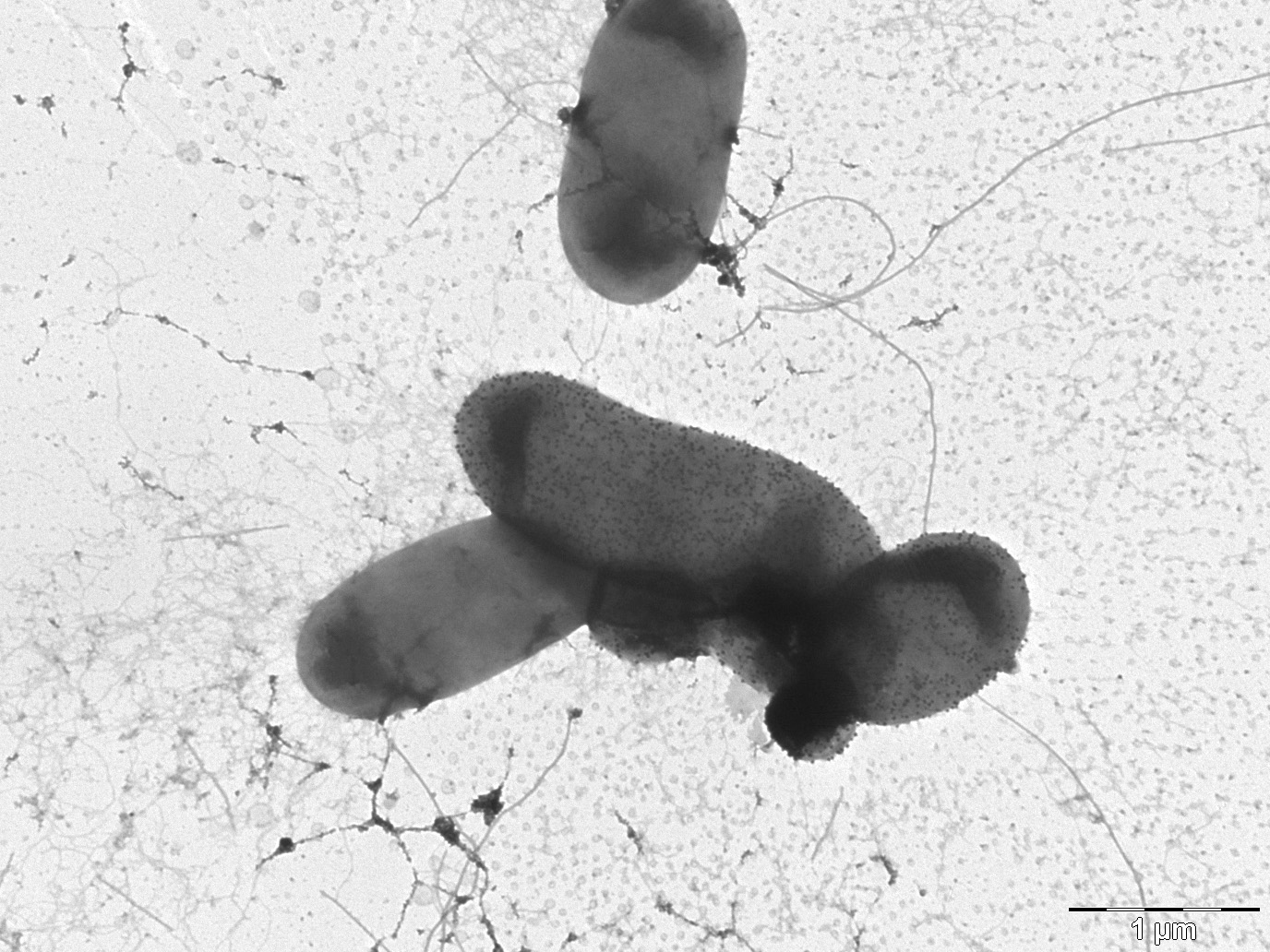

Supplement: Figure 7—source data 1. [file elife-95328-fig7-data1.zip › imagenes figura 7/SV5015-R27-dil800-7.jpg]

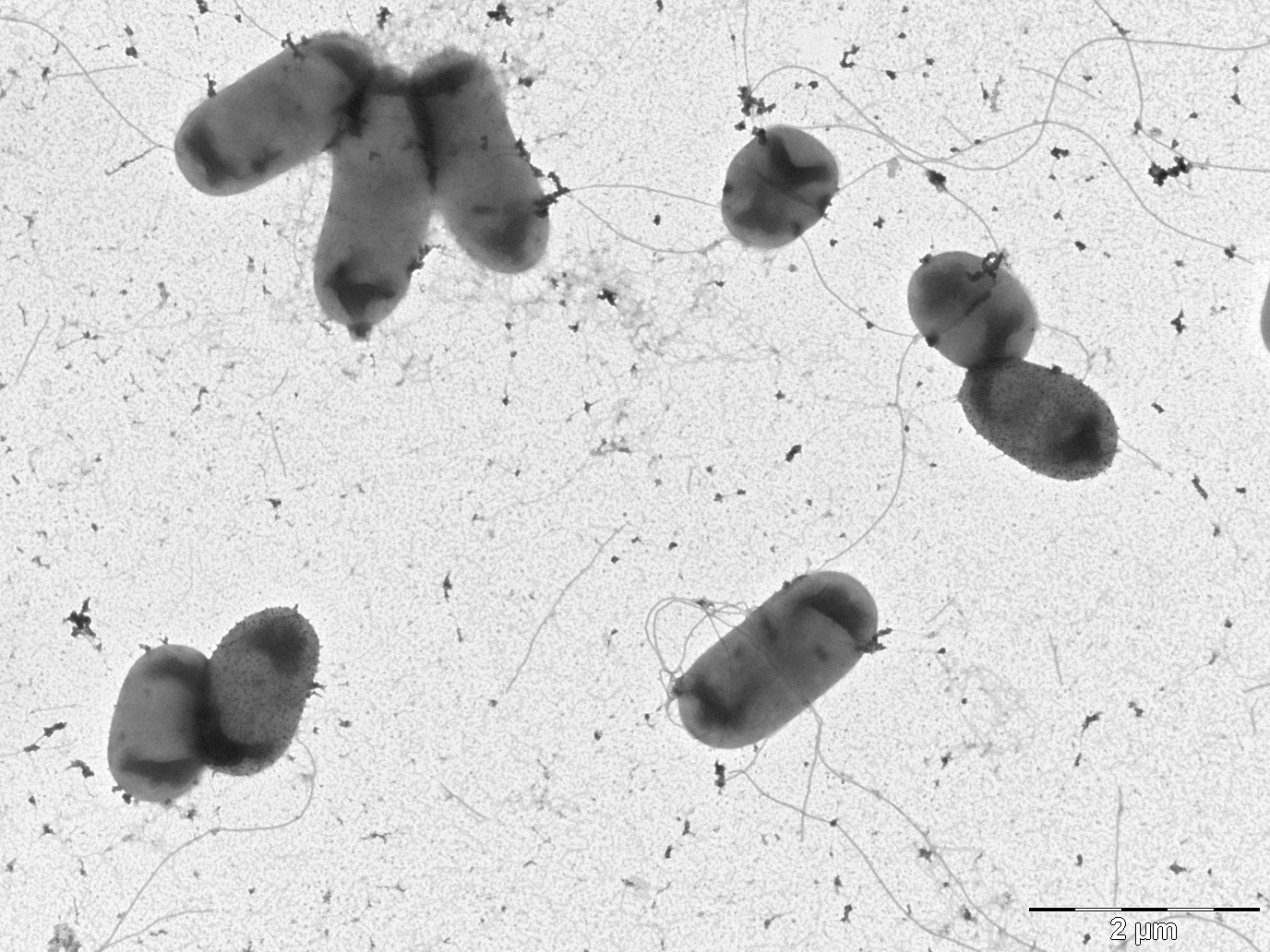

Supplement: Figure 7—source data 1. [file elife-95328-fig7-data1.zip › imagenes figura 7/SV5015-R27-dil800-10.jpg]

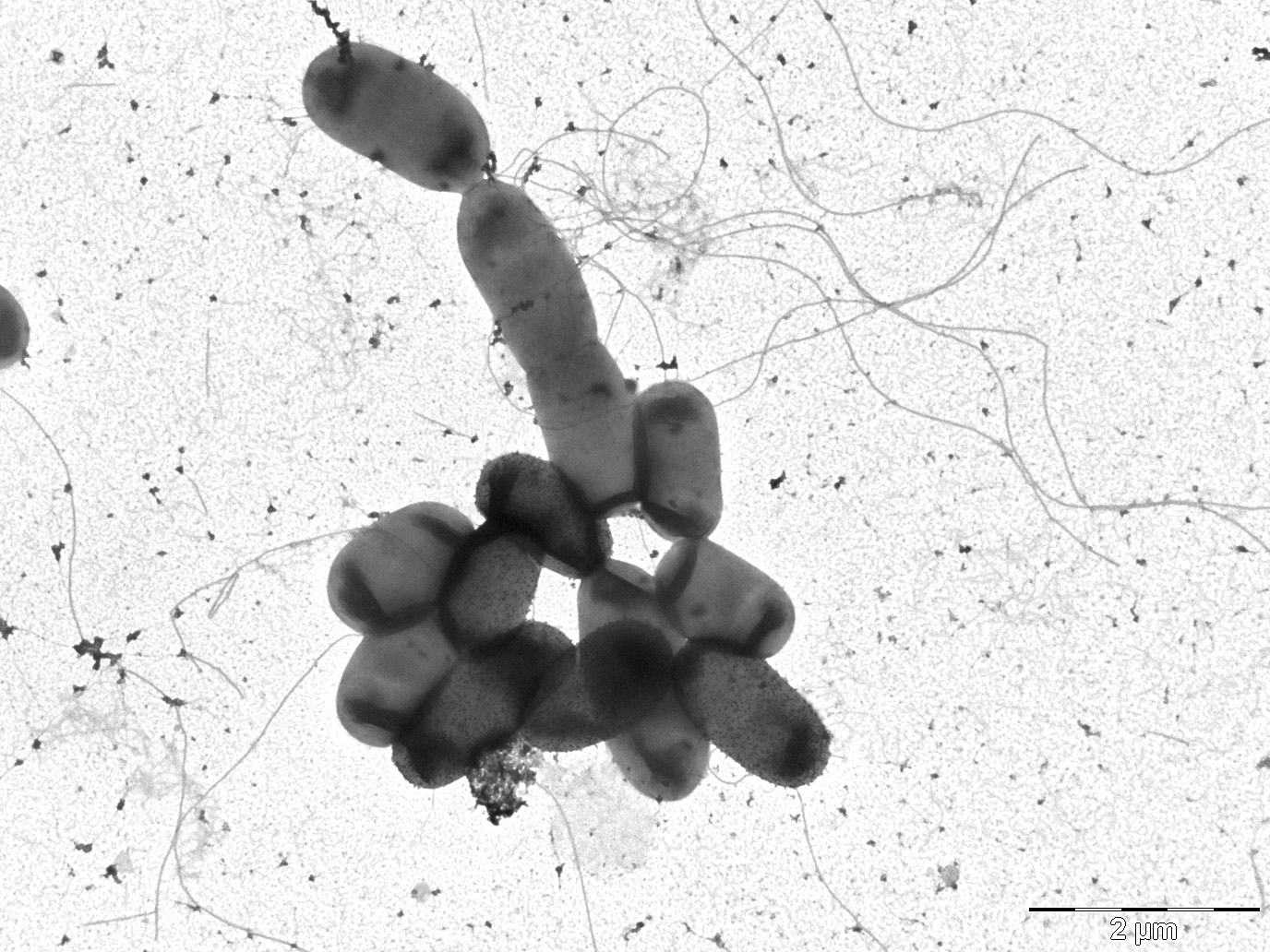

Supplement: Figure 7—source data 1. [file elife-95328-fig7-data1.zip › imagenes figura 7/SV5015-R27-dil800-11.jpg]

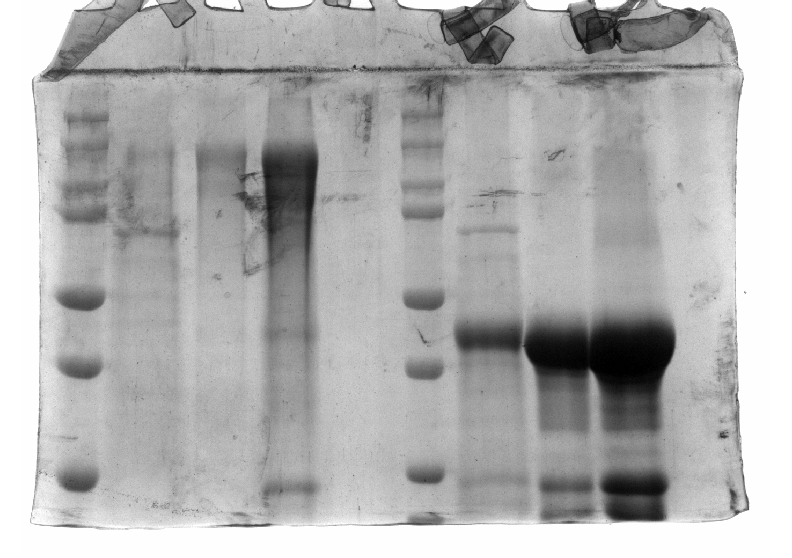

Supplement: Figure 8—source data 1. [file elife-95328-fig8-data1.zip › Figure 8-source data 1.tif]
