# Supplementary material for: Targeting plasmid-encoded proteins that contain immunoglobulin-like domains to combat antimicrobial resistance
Source: eLife. 2024 Jul 24;13:RP95328. doi: 10.7554/eLife.95328 (PMC11268884; doi:10.7554/eLife.95328)
Supplement: Figure 8—source data 2. [file elife-95328-fig8-data2.pdf]

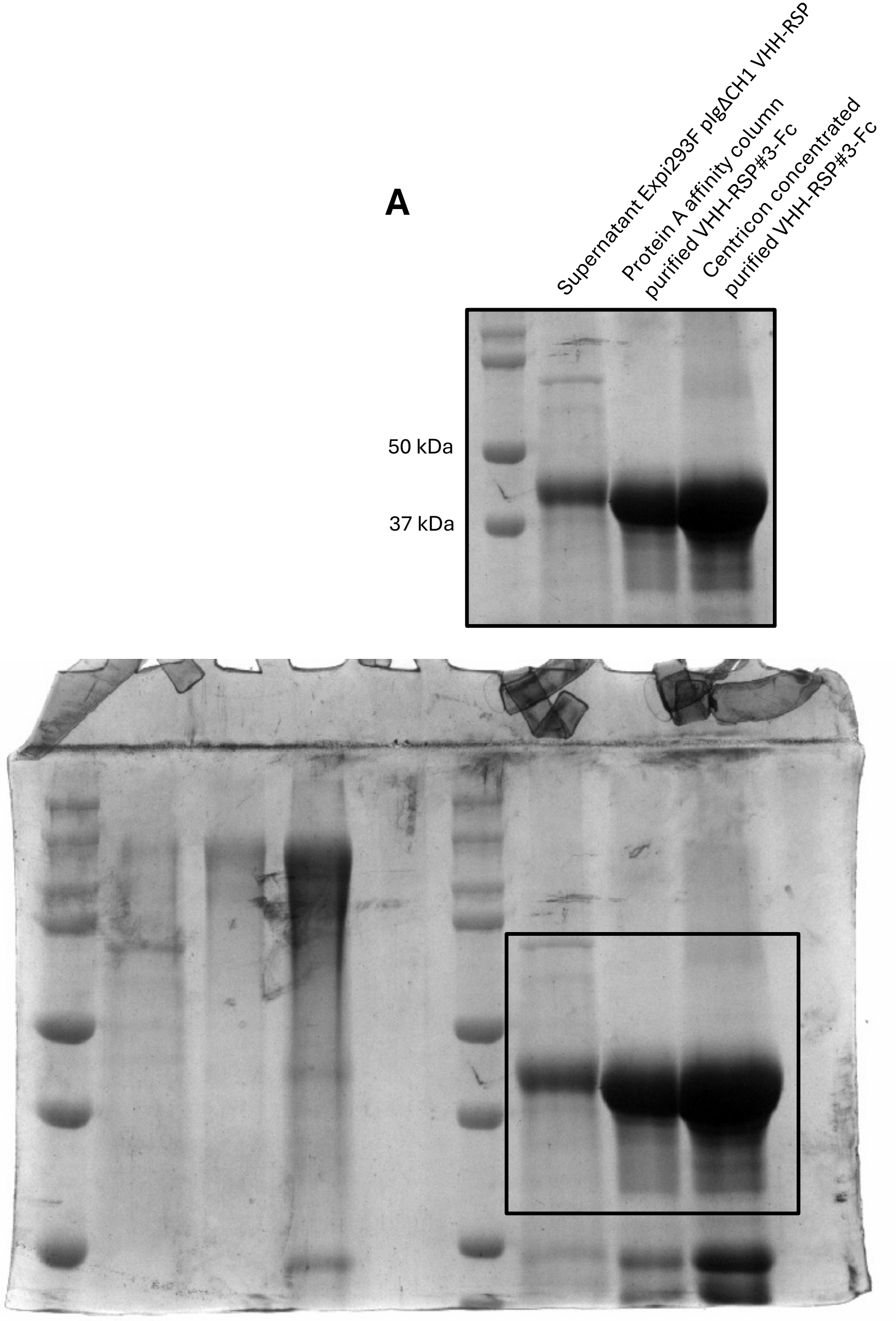
A
Supernatant Expi293F pIgΔCH1 VHH-RSP
Protein A affinity column purified VHH-RSP#3-Fc
Centricon concentrated purified VHH-RSP#3-Fc
50 kDa
37 kDa
