# Supplementary material for: Targeting plasmid-encoded proteins that contain immunoglobulin-like domains to combat antimicrobial resistance
Source: eLife. 2024 Jul 24;13:RP95328. doi: 10.7554/eLife.95328 (PMC11268884; doi:10.7554/eLife.95328)
Supplement: Supplementary file 1. — (a) List of the strains and plasmids used in this work. (b) List of the oligonucleotides used in this work. [file elife-95328-supp1.docx]

**Supplementary Material**

Targeting plasmid-encoded proteins that contain immunoglobulin-like domains to combat antimicrobial resistance.

Alejandro Prieto^1*^, Lluïsa Miró^2,3*^, Yago Margolles^4^, Manuel Bernabeu^1^, David Salguero^1^, Susana Merino^1^, Joan Tomás^1^, Juan Alberto Corbera^5^, Anna Pérez-Bosque^2,3^, Mario Hüttener^1^, Luis Ángel Fernández^4^ and Antonio Juárez^1,6, **^

1. Department of Genetics, Microbiology and Statistics, University of Barcelona, Barcelona, Spain.

2. Department of Biochemistry and Physiology, Universitat de Barcelona, Barcelona, Spain.

3. Institut de Nutrició i Seguretat Alimentària, Universitat de Barcelona, Barcelona, Spain.

4. Department of Microbial Biotechnology, Centro Nacional de Biotecnología, Consejo Superior de Investigaciones Científicas (CNB-CSIC), Madrid, Spain.

5. Instituto Universitario de Investigaciones Biomédicas y Sanitarias (IUIBS), Facultad de Veterinaria, Universidad de Las Palmas de Gran Canaria (ULPGC), Campus Universitario de Arucas, Las Palmas, Spain.

6. Institute for Bioengineering of Catalonia, The Barcelona Institute of Science and Technology, Barcelona, Spain.

*Both authors contributed equally to this work.

**Corresponding author: Prof. Antonio Juárez, [ajuarez@ub.edu](mailto:ajuarez@ub.edu)

**Supplementary File 1a**. List of the strains and plasmids used in this work.

| Strain | Genotype and relevant properties | Reference |
| --- | --- | --- |
| *E. coli* DH10BT1R | F- *mcrA* Δ*mrr-hsdRMS-mcrBC* φ80lacZDM15 Δ*lacX74 recA1 endA1 araD139* Δ(*ara,leu*) *7697 galU galK rpsL (StrR) nupG tonA λ* | Invitrogen |
| *E. coli* BL21DE3 | *hsdS, gal, (λclts857, ind1, Sam7, nin5, lac-UV5-T7gene1)* | (Studier and Moffatt, 1986) |
| *S.* Typhimurium SL1344 | *rspLhisG* | (Hoiseth and Stocker, 1981) |
| *S.* Typhimurium SL1344 *ibpA::lacZ-Kmr* | *rspLhisG ibpA::lacZ-Km^r^* | (Hüttener et al., 2018) |
| *S.* Typhimurium SL1344 *flhDC::Km* | *rspLhisG flhDC::Km* | This work |

| Plasmid | Description | Reference |
| --- | --- | --- |
| R27 | IncHI1, Tc^r^ | (Grindley et al., 1972) |
| R27 Δ*rsp* | R27 *rsp*::FRT | (Hüttener et al., 2019) |
| pHCM1 | IncHI1, Amp^r^ | (Parkhill et al., 2001) |
| pKD4 | *bla* (Ap^r^) FRT *ahp* FRT PS1 PS2 oriR6K Km^r^ | (Datsenko and Wanner, 2000) |
| pKD46 | *bla* (Ap^r^) P_BAD_ *gam bet exo* pSC101 oriTS | (Datsenko and Wanner, 2000) |
| pNeae2 | pNeae-derivative; for fusions to Neae-myc [Intimin_EHEC_ (1-654)-E-His-myc tag | (Salema et al., 2013) |
| pNeae2 VHH-RSP #3 | VHH against RSP protein fused to Neae-myc [Intimin_EHEC_ (1-654)-E-His-myc tag | This work |
| pNVFib1 | pNeae-myc-derivative; NVFib (clone 1) fusion [Intimin_EHEC_ (1-654)-E-VFIBn-myc tag] | (Salema et al., 2016) |
| pMAL-RSP #5/7 | pMAL-p2E derivative, for fusion of C-terminal of RSP to MBP | This work |
| pIgΔCH1 | pIgγ1HC derivative vector lacking human IgG CH1 domain | (Casasnovas et al., 2022) |
| pIgΔCH1 VHH-RSP | IgH signal peptide, VHH-RSP fused to the human IgG1 hinge and Fc portion (Fc) | This work |

**Supplementary File 1b**. List of the oligonucleotides used in this work.

| Oligonucleotide | Sequence (5’-3’) |
| --- | --- |
| RSP5 BamHI Fw | 5’ CGGGATCCTGGGGTGTATAAGTTTCCT 3’ |
| RSP PstI Rv | 5’ AACTGCAGTTACTGCGAGGTTTCAAC 3’ |
| flhDC p1 | 5’GGCTACGTCGCACAAAAATAAAGTTGGTTATTCTGGATGGGAGTGTAGGCTGGAGCTGCTTC 3’ |
| flhDC p2 | 5’TTACCGCTGCTGGAGTGTTTGTCCACACCGTTTCGGTTAAACCATATGAATATCCTCCTTAGT 3’ |
| flhDC p1 up | 5’ CGTTGTATGTCACGAAGCTGAC 3’ |
| flhDC p2 down | 5’ GCTGTTGACTATGACAGGATGC 3’ |
| VHH Sfi | 5’ GTCCTCGCAACTGCGGCCCAGCCGGCCATGGCTCAGGTGCAGCTGGTG GA 3’ |
| VHH Not | 5’ GGACTAGTGCGGCCGCTGAGGAGACGGTGACCTGGGT 3’ |
| VHH pIg AgeI | 5’ ACTGCAACCGGTGTACATTCTCAGGTGCAGCTGGTGGA 3’ |
| VHH pIg BamHI | 5’ ACCGGATCCACGCGGAACCAGCGCTGAGGAGACGGTGACCTG 3’ |
| *Il-1* β Fw | 5’ GGTCAAAGGTTTGGAAGCAG 3’ |
| *Il-1* β Rv | 5’ TGTGAAATGCCACCTTTTGA 3’ |
| *Il*-6 Fw | 5’ TGTGAAATGCCACCTTTTGA 3’ |
| *Il*-6 Rv | 5’ GGTCAAAGGTTTGGAAGCAG 3’ |
| *Tnf-α* Fw | 5’ CCACCACGCTCTTCTGTCTAC 3’ |
| *Tnf-α* Rv | 5’ AGGGTCTGGGCCATAGAACT 3’ |
| *Hprt1* Fw | 5’ TGGATACAGGCCAGACTTTGTT 3’ |
| *Hprt1* Rv | 5’ CAGATTCAACTTGCGCTCATC 3’ |
